# Supplementary material for: Heat adaptation of phage T7 under an extended genetic code
Source: Virus Evol. 2021 Dec 1;7(2):veab100. doi: 10.1093/ve/veab100 (PMC8923235; doi:10.1093/ve/veab100)
Supplement: veab100_Supp [file veab100_supp.zip › Sequencing_Analysis.zip!/Sequencing_Analysis/S3/evidence/JC_SIDE_1_47.html]

BRESEQ :: Evidence


| New junction evidence | | | | | | | | | | | |
| --- | --- | --- | --- | --- | --- | --- | --- | --- | --- | --- | --- |
|  | | seq id | position | reads (cov) | reads (cov) | score | skew | freq | annotation | gene | product |
| \* | ? | NC\_001604.1 | = 7972 | 2034 (1.180) | 21 (0.010) | 20/588 | 34.7 | 1.1% | intergenic (–/–) | *–/–* | –/– |
|  | ? | NC\_001604.1 | 8524 = | 1958 (1.160) |  |  |  |  | intergenic (–/–) | *–/–* | –/– |
| Rejected: Coverage evenness skew score above cutoff. | | | | | | | | | | | |
| Rejected: Frequency below/above cutoff threshold. | | | | | | | | | | | |

Only 100 of 2050 total aligned reads displayed.  

`TTGCGGTATACCCTCAAGTAGCACTAGTAGTAGTTGGCGCTTGTTACTTAGCGGCAGTGTGTGCTTGCGTGTGGAGTATAGTTAACTGGTAATACGACTCACTAAAGGAGGTACACACCATGATGTACTTAATGCCATTACTCATCGTCATTGTAGGATGCCTTGCGCTCCACTGTAGCGATGATGATATGCCAGATGGTCACGCTTAATACGACTCACTAAAGGAGACACTATATGTTTCGACTTCATTACAACAAAAGCGTTAAGAATTTCACGGTTCGCCGTGCTGACCGTTCAATCGTATGTGCGAGCGAGCGCCGAGCTAAGATACCTCTTATTGGTAACACAGTTCCTTTGGCACCGAGCGTCCACATCATTATCACCCGTGGTGACTTTGAGAAAGCAATAGACAAGAAACGTCCGGTTCTTAGTGTGGCAGTGACCCGCTTCCCGTTCGTCCGTCTGTTACTCAAACGAATCAAGGAGGTGTTCTGATGGGACTGTTAGATGGTGAAGCCTGGGAAAAAGAAAACCCGCCAGTACAAGCAACTGGGTGTATAGCTTGCTTAGAGAAAGATGACCGTTATCCACACACCTGTAA  >  NC_001604.1/7672‑8272`  

                                                                                                                                                                                                                                                                                                            `|`                                                                                                                                                                                                                                                                                                              
   `cGGTATACCCTCAAGTAGCACTAGTAGTAGTTGGCGCTTGTTACTTAGCGGCAGTGTGTGCTTGCGTGTGGAGTATAGTTAACTGGTAATACGACTCACTAAAGGAGGTACACACCATGATGTACTTAATGCCATTACTCATCGTCATTGTAGGATGCCTTGCGCTCCACTGTAGCGATGATGATATGCCAGATGGTCACGCTTAATACGACTCACTAAAGGAGACACTATATGTTTCGACTTCATTACAACAAAAGCGTTAAGAATTTCACGGTTCGCCGTGCTGACCGTTCAATCGTAt                                                                                                                                                                                                                                                                                                           <  1:139562/301‑1 (MQ=255)`  
   `cGGTATACCCGCAATGAGCACTAGTAGGAGTGGGCGCGTGTTACTTAGCGGCAGTGTGTGCTTGCGTGTGGAGTATAGTTAACTGGTAATACGACTCACTAAAGGAGGTACACACCATGTTGTACTTAATGCCATTACTCATCGTCATTGTAGGATGCCTTGCGCTCCACTGTAGCGATGATGATATGCCAGATGGTCACGCTTAATACGACTCACTAAAGGAGACACTATATGTTTCGACTTCATTACAACAAAAGCGTTAAGAATTTCACGGTTCGCCGTGCTGACCGTTCAATCGTAt                                                                                                                                                                                                                                                                                                           <  1:75569/301‑1 (MQ=255)`  
     `gTATACCCTCAAGTAGCACTAGTAGTAGTTGGCGCTTGTTACTTAGCGGCAGTGTGTGCTTGCGTGTGGAGTATAGTTAACTGGTAATACGACTCACTAAAGGAGGTACACACCATGATGTACTTAATGCCATTACTCATCGTCATTGTAGGATGCCTTGCGCTCCACTGTAGCGATGATGATATGCCAGATGGTCACGCTTAATACGACTCACTAAAGGAGACACTATATGTTTCGACTTCATTACAACAAAAGCGTTAAGAATTTACCGGTTCGCCGTGCTGACCGTTCAATCGTAtgt                                                                                                                                                                                                                                                                                                         >  1:34801/1‑301 (MQ=255)`  
      `tataCCCTAAAGTAGCACTAGTAGTAGTGTGCGCTGGTTACTTAGCGGCAGGGTGTGCTTGCGTGTGGAGTATAGTTAACTGGTAATACGACTCACTAAAGGAGGTACACACCATGATGTACTTAATGCCATTACTCATCGTCATTGTAGGATGCCTTGCGCTCCACTGTAGCGATGATGATATGCCAGATGGTCACGCTTAATACGACTCACTAAAGGAGACACTATATGTTTCGACTTCATTACAACAAAAGCGTTAAGAATTTCACGGTTCGCCGTGCTGACCGTTCAATCGTAtgtg                                                                                                                                                                                                                                                                                                        <  1:153768/301‑1 (MQ=255)`  
          `cccTCAAGTAGCACTCTTCGTCGTTGGCGCTTGTTACTTAGCGGCAGTGTGTGCTTGCGTGTGGAGTATAGTTAACTGGTAATACGACTCACTAAAGGAGGTACACACCATGATGTACTTAATGCCATTACTCATCGTCATTGTAGGATGCCTTGCGCTCCACTGTAGCGATGATGATATGCCAGATGGTCACGCTTAATACGACTCACTAAAGGAGACACTATATGTTTCGACTTCATTACAACAAAAGCGTTAAGAATTTCACGGTTCGCCGTTCTGACCGTTCAATCGTATGTccgag                                                                                                                                                                                                                                                                                                    >  1:167464/1‑301 (MQ=255)`  
          `cccTCAAGTAGCACTAGTAGTAGTTGGCGCTTGTTACTTAGCGGCAGTGTGTGCTTGCGTGTGGAGTATAGTTAACTGGTAATACGACTCACTAAAGGAGGTACACACCATGATGTACTTAATGCCATTACTCATCGTCATTGTAGGATGCCTTGCGCTCCACTGTAGCGATGATGATATGCCAGATGGTCACGCTTAATACGACTCACTAAAGGAGACACTATATGTTTCGACTTCATTACAACAAAAGCGTTAAGAATTTCACGGTTCGCCGTGCTGACCGTTCAATCGTATGTgcgag                                                                                                                                                                                                                                                                                                    >  1:97395/1‑301 (MQ=255)`  
                       `cTAGTAGTAGTTGGCGCTTGTTACTTAGCGGCAGTGTGTGCTTGCGTGTGGAGTATAGTTAACTGGTAATACGACTCACTAAAGGAGGTACACACCATGATGTACTTAATGCCATTACTCATCGTCATTGTAGGATGCCTTGCGCTCCACTGTAGCGATGATGATATGCCAGATGGTCACGCTTAATACGACTCACTAAAGGAGACACTATATGTTTCGACTTCATTACAACAAAAGCGTTAAGAATTTCACGGTTCGCCGTGCTGACCGTTCAATCGTATGTGCgagcgagcctgtctct                                                                                                                                                                                                                                                                                       >  1:100130/1‑293 (MQ=255)`  
                            `aagagacagcgcTTGGTACTTAGCGGCAGTGTGAGCTTGCGTGTGGAATATAGTTAACTGGTAATACGACTCACTAAAGGAGGTACACACCATTATGTACTTAATGCCATTACTCATCGTCATTGTAGGATGCCTTGCGCTCCACTGTAGCGATGATGATATGCCAGATGGTCACGCGTAATACGACTCACTAAAGGAGACACTATATGTTTCGACTTCAGTACAACAAAAGCGTTAAGAATTTCACGGTTCGCCGTGCTGACCTTTCAATCGTATGTGCGAGCGAGCGCCGAGCTAAGAt                                                                                                                                                                                                                                                                                  <  1:238756/293‑1 (MQ=255)`  
                                   `ggCGCTTGTTACTTAGCGGCAGTGTGTGCTTGCGTGTGGAGTATAGTTAACTGGTAATACGACTCACTAAAGGAGGTACACACCATGATGTACTTAATGCCATTACTCATCGTCATTGTAGGATGCCTTGCGCTCCACTGTAGCGATGATGATATGCCAGATGGTCACGCTTAATACGACTCACTAAAGGAGACACTATATGTTTCGACTTCATTACAACAAAAGCGTTAAGAATTTCACGGTTCGCCGTGCTGACCGTTCAATCGTATGTGCGAGCGAGCGCCGAGCTAAGATACCCCtt                                                                                                                                                                                                                                                                           >  1:121846/1‑301 (MQ=255)`  
                                    `gcgcTTGTTACTTAGCGGCAGTGTGTGCTTGCGTGTGGAGTATAGTTAACTGGTAATACGACTCACTAAAGGAGGTACACACCATGATGTACTTAATGCCATTACTCATCGTCATTGTAGGATGCCTTGCGCTCCACTGTAGCGATGATGATATGCCAGATGGTCACGCTTAATACGACTCACTAAAGGAGACACTATATGTTTCGACTTCATTACAACAAAAGCGTTAAGAATTTCACGGTTCGCCGTGCTGACCGTTCAATCGTATGTGCGAGCGAGCGCCGAGCTAAGATACCTCttt                                                                                                                                                                                                                                                                          >  1:7890/1‑300 (MQ=255)`  
                                      `gcTTGATACTTAGCGGCAGTGTTTGCTTGCGTGTGGAGTATAGTTAACTGGTAATACGACTCACTAAAGGAGGTACACACCATGATGTACTTAATGCCATTACTCATCGTCATTGTAGGATGCCTTGCGCTCCACTGTAGCGATGATGATATGCCAGATGGTCACGCTTAATACGACTCACTAAAGGAGACACTATATGTTTCGACTTCATTACAACAAAAGCGTTAAGAATTTCACGGTTCGCCGTGCTGACCGTTCAATCGTATGTGCGAGCGAGCGCCGAGCTAAGATACCTCTTAtt                                                                                                                                                                                                                                                                        <  1:142332/301‑1 (MQ=255)`  
                                                        `gtgtgtGCTTGCGTGTGGAGTATAGTTAACTGGTAATACGACTCACTAAAGGAGGTACACACCATGATGTACTTAATGCCATTACTCATCGTCATTGTAGGATGCCTTGCGCTCCACTGTAGCGATGATGATATGCCAGATGGTCACGCTTAATACGACTCACTAAAGGAGACACTATATGTTTCGACTTCATTACAACAAAAGCGTTAAGAATTTCACGGTTCGCCGTGCTGACCGTTCAATCGTATGTGCGAGCGAGCGCCGAGCTAAGATACCTCTTAttctgtctcttatacacatc                                                                                                                                                                                                                                                      >  1:43435/1‑283 (MQ=255)`  
                                                        `gtgtgtGCTTGCGTGTGGAGTATAGTTAACTGGTAATACGACTCACTAAAGGAGGTACACACCATGATGTACTTAATGCCATTACTCATCGTCATTGTAGGATGCCTTGCGCTCCACTGTAGCGATGATGATATGCCAGATGGTCACGCTTAATACGACTCACTAAAGGAGACACTATATGTTTCGACTTCATTACAACAAAAGCGTTAAGAATTTCACGGTTCGCCGTGCTGACCGTTCAATCGTATGTGAGAGCGAGCGCCGAGCTAAGATACCTCTTATTGGTAACACAGTTCCTTTg                                                                                                                                                                                                                                                      >  1:88050/1‑301 (MQ=255)`  
                                                        `gtgtgtGCTTGCGTGTGGAGTATAGTTAACTGGTAATACGACTCACTAAAGGAGGTACACACCATGATGTACTTAATGACATTACTCATCATCCTTGTATGATGCCTTTCGCTACACTGTAGCGATGATGATATGCCAGATAGTCACGCTTAATACGACTCACTAAATGAGACAATATATGTTTCGACTTCATTACCACAAAAGCGTTACGAATTTCACGGTTCCCCGTTCAGACCGTTCAATCGTATGTTCGATCGAGTGCCGCGCTAAGCTCCCTCTTATTGGTACCACATTTCCTTTg                                                                                                                                                                                                                                                      >  1:36992/1‑301 (MQ=255)`  
                                                            `gtgCTTGCGTGTGGAGTATAGTTAACTGGTAATACGACTCACTAAAGGAGGTACACACCATGATGTACTTAATGCCATTACTCATCGTCATTGTAAGATGCCTTGCGCTCCACTGTAGCGATGATGATATGCCAGATGGTCACGCTTAATACGACTCACTAAAGGAGACACTATATGTTTCGACTTCATTACAACAAAAGCGTTAAGAATTTCACGGTTCGCCGTGCTGACCGTTCAATCGTATGTGCGAGCGAGCGCCGAGCTAAGATACCTCTTATTGGTAACACAGTTCCTTTGGCAc                                                                                                                                                                                                                                                  <  1:60991/301‑1 (MQ=255)`  
                                                                        `ggAGTATAGTTAACTGGTAATACGACTCACTAAAGGATGTACACACCACGATGTACTTAATGCCATTACTCATCGTCATTGTAGGATGCCTTGCGCTCCACTGTAGCGATGATGATATGCCAGATGGTCACGCTTAATACGACTCACTAAAGGAGACACTATATGTTTCGACTTCATTACAACAAAAGCGTTAAGAATTTCACGGTTCGCCGTGCTGACCGTTCAATCGTATGTGCGAGCGAGCGCCGAGCTAAGATACCTCTTATTGGTAACACAGTTCCTTTGGctgtctcttataaac                                                                                                                                                                                                                                      >  1:157891/1‑287 (MQ=255)`  
                                                                         `gAGTATAGTTAACTGGTAATACGACTCACTAAAGGAGGTACACACCATGATGTACTTAATGCCATTACTCATCGTCATTGTAGGATGCCTTGCGCTCCACTGTAGCGATGATGATATGCCAGATGGTCACGCTTAATACGACTCACTAACGGAGACACTATATGTTTCGACTTCATTACAACAAAAGCGTTAAGAATTTTACGGTTCGCCGTGCTGACCGTTCAATCGTATGTGCGAGCGAGCGCCGAGCTAAGATACCTCTTATTGGTAACACAGTTCCTTTGGCACCGGGCGTCCAcat                                                                                                                                                                                                                                     >  1:196941/1‑301 (MQ=255)`  
                                                                         `gAGGATAGTTAACTGGTAATACGACTCACTAAAGGAGGAACACACCATGATGTACTTAATGCGATTACTCAACGTCATTGTAGGATGCCTTGCGCTCAAATGTAGCGATGATGATATGCGAGATGGTCACGCTTAATCCGACTCACTAAAGGAGACACTATATGTTTAGTCTTCATTAAAACAAAAGCGTTAAGAATTTCACGGATAGACGTGCTGACCGTTCAATCGTATGTGCGAGCGAGCGCCGAGCTAAGATACCTCTTATTGGTAAAAAAGTTCCTTTGGCACCGAGCGTCCAcat                                                                                                                                                                                                                                     >  1:144411/1‑301 (MQ=255)`  
                                                                             `ataGTTAACTGGTAATACGACTCACTAAAGGAGGTACACACCATGATGTACTTAATGCTATTACTCATCGTCATTGTAGGATGCATTGCGATCCACTGTAGCGATGATGATATGCCAGATGGTAACGCTTAATACGACTCACTCAAGGAGACACTATATGTTTCGACTTCATTATAACAAAATCGTTAAGAATTTCTTGGTTCGCAGTGCTGATCGTTCAATCGTATGTGTGAGCGAGCGCCTAGCTAAGATAACTCTTATTGGTAACACAATTTCTTTGTCatcaagaagttacatattt                                                                                                                                                                                                                                 >  1:71878/1‑283 (MQ=255)`  
                                                                             `ataGTTAACTGGTAATACGACTCACTAAAGGAGGTACACACCATGATGTACTTAATGCCATTACTCATCGTCATTGTAGGATGCCTTGCGCTCCACTGTAGCGATGATGATATGCCAGATGGTCACGCTTAATACGACTCACTAAAGGAGACACTATATGTTTCGACTTCATTACAACAAAAGCGTTAAGAATTTCACGGTTCGCCGTGCTGACCGTTCAATCGTATGTGCGAGCGAGCGCCGAGCTAAGATACCTCTTATTGGTAACACAGTTccctgtctcttatacacatctccgagc                                                                                                                                                                                                                                 >  1:112708/1‑276 (MQ=255)`  
                                                                                 `acagcTGGTAATACGACTCACTAAAGGAGGTACACACCATGATGTACTTAATGCCATTACTCATCGTCATTGTAGGATGCCTTGCGCTCCACGGTAGCGATGATGATATGCCAGATGGTCACGCTTAATACGACTCACTAAAGGAGACACTATATGTTTCGACTTCATTACAACAAAAGCGTTAAGAATTTCACGGTTCGCCGTGCTGACCGTTCAATCGTATGTGCGAGCGAGCGCCGAGCTAAGATACCTCTTATTGGTAACACAGTTCCTTTGGCACCGAGCGTCCACATCATTATCa                                                                                                                                                                                                                             <  1:236674/297‑1 (MQ=255)`  
                                                                                   `aaCTGGTAATACGACTCACTAAAGGAGGTACACACCATGATGTACTTAATGCCATTACTCATCGTCATTGTAGGATGCCTTGCGCTCCACTGTAGCGATGATGATATGCCAGATGGTCACGCTTAATACGACTCACTAAAGGAGACACTATATGTTTCGACTTCATTACAACAAAAGCGTTAAGAATTTCACGGTTCGCCGTGCTGACCGTTCAATCGTATGTGCGAGCGAGCGCCGAGCTAAGATACCTCTTATTGGTAACACAGTTCCTTTGGCACCGAGCGTCTACATCATTATCAcc                                                                                                                                                                                                                           <  1:263351/301‑1 (MQ=255)`  
                                                                                       `ggTAATACGACTCACTAAAGGAGGTACACACCATGATGTACTTAATGCCATTACTCATCGTCATTGTAGGATGCCTTGCGCTCCACTGTAGCGATGATGATATGCCAGATGGTCACGCTTAATACGACTCACTAAAGGAGACACTATATGTTTCGACTTCATTACAACAAAAGCGTTAAGAATTTCACGGTTCGCCGTGCTGACCGTTCAATCGTATGTGCGAGCGAGCGCTGAGCTAAGATACCTCTTATTGGTAACACAGTTCCTTTGGCACCTATCGTCCACATCATTATCACCCgtg                                                                                                                                                                                                                       >  1:207115/1‑301 (MQ=255)`  
                                                                                        `gTAATACGACTCACTAAAGGAGGTACACACCATGATGTACTTAATGCCATTACTCATCGTCATTGTAGGATGCCTTGCGCTCCACTGTAGCGATGATGATATGCCAGATGGTCACGCTTAATACGACTCACTAAAGGAGACACTATATGTTTCGACTTCATTACAACAAAAGCGTTAAGAATTTCACGGTTCGCCGTGCTGACCGTTCAATCGTATGTGCGAGCGAGCGCCGAGCTAAGATACCTCTTATTGGTAACACAGTTCCTTTGGCACCGAGCGTCCACATCATTATCACCCgtgg                                                                                                                                                                                                                      >  1:115162/1‑301 (MQ=255)`  
                                                                                                   `cACTAAAGGAGGTACACACCATGATGTACTTAATGCCATTACTCATCGTCGTTGTAGGATGCCTTGCGCTCCACTGTAGCGATGATGATATGCCAGATGGTCACGCTTAATACGACTCACTAAAGGAGACACTATATGTTTCGACTTCATTACAACAAAAGCGTTAAGAATTTCACGGTTCGCCGTGCTGACCGTTCAATCGTATGTGCGAGCGAGCGCCGAGCTAAGATACCTCTTATTGGTAACACAGTTCCTTTGGCACCGAGCGTCCACATCATTATCACCCctgtctcttatacac                                                                                                                                                                                                           >  1:133096/1‑289 (MQ=255)`  
                                                                                                    `gtgtataagagacagcacCATGATGTACTTAATGCCATTACTCATCGTCATTGTAGGATGCCTTGCGCTCCACTGTAGCGATGATGATATGCCAGATGGTCACGCTTAATACGACTCACTAAAGGAGACACTATATGTTTCGACTTCATTACAACAAAAGCGTTAAGAATTTCACGGTTCGCCGTGCTGACCGTTCAATCGTATGTGCGAGCGAGCGCCGAGCTAAGATACCTCTTATTGGTAACACAGTTCCTTTGGCACCGAGCGTCCACATCATTATCACCCGTGGTGACTTTGAGaa                                                                                                                                                                                                          <  1:102488/286‑1 (MQ=255)`  
                                                                                                    `gcTAAAGGAGGTACACACCATGATGTACTTAATGCCATTACTCATCGTCATTGTAGGATGCCTTGCGCTCCACTGTAGCGATGATGATATGCCAGATGGTCACGCTTAATACGACTCACTAAAGGAGACACTATATGTTTCGACTTCATTACAACAAAAGCGTTAAGAATTTCACGGTTCGCCGTGCTGACCGTTCAATCGTATGTGCGAGCGAGCGCCGAGCTAAGATACCTCTTATTGGTAACACAGTTCCTTTGGCACCGAGCGTCCACATCATTATCACCCGTGGTGACTTTGAGaa                                                                                                                                                                                                          <  1:127093/300‑1 (MQ=255)`  
                                                                                                    `aagagacagggTACACACCATGATGTACTTAATGCCATTACTCATCGTCATTGTAGGATGCCTTGCGCTCCACTGTAGCGATGATGATATGCCAGATGGTCACGCTTAATACGACTCACTAAAGGAGACACTATATGTTTCGACTTCATTACAACAAAAGCGTTAAGAATTTCACGGTTCGCCGTGCTGACCGTTCAATCGTATGTGCGAGCGAGCGCCGAGCTAAGATACCTCTTATTGGTAACACAGTTCCTTTGGCACCGAGCGTCCACATCATTATCACCCGTGGTGACTTTGAGaa                                                                                                                                                                                                          <  1:161350/292‑1 (MQ=255)`  
                                                                                                          `ggaggTACACACCATGATGTACTTAATGCCATTACTCATCGTCATTGTAGGATGCCTTGCGCTCCACTGTAGCGATGATGATATGCCAGATGGTCACGCTTAATACGACTCACTAAAGGAGACACTATATGTTTCGACTTCATTACAACAAAAGCGTTAAGAATTTCACGGTTCGCCGTGCTGACCGTTCAATCGTATGTGCGAGCGAGCGCCGAGCTAAGATACCTCTTATTGGTAACACAGTTCCTTTTTCACCGAGCGTCCACATCATTATcctgtctcttatacacatctccgagtc                                                                                                                                                                                                    >  1:46954/1‑275 (MQ=255)`  
                                                                                                              `gTACACACCATGATGTACTTAATGCCATTACTCATCGTCATTGTAGGATGCCTTGCGCTCCACTGTAGCGATGATGATATGCCAGATGGTCACGCTTAATACGACTCACTAAAGGAGACACTATATGTTTCGACTTCATTACAACAAAAGCGTTAAGAATTTCACGGTTCGCCGTGCTGACCGTTCAATCGTATGTGCGAGCGAGCGCCGAGCTAAGATACCTCTTATTGGTAACACAGTTCCTTTGGCACCGAGCGTCCACATCATTATCACCCGTGGTGACTTTGAGAAAGCAATAGAc                                                                                                                                                                                                <  1:311450/301‑1 (MQ=255)`  
                                                                                                                     `ccATGATGTACTTAATGCCATTACTCATCGTCATTGTAGGATGCCTTGCGCTCCACTGTAGCGATGATGATATGCCAGATGGTCACGCTTAATACGACTCACTAAAGGAGACACTATATGTTTCGACTTCATTACAACAAAAGCGTTAAGAATTTCACGGTTCGCCGTGCTGACCGTTCAATCGTATGTGCGAGCGAGCGCCGAGCTAAGATACCTCTTATTGGTAACACAGTTCCTTTGGCACCGAGCGTCCACATCATTATCACCCGTGGTGACTTTGAGAAAGCAATAGACAAGAAAc                                                                                                                                                                                         >  1:314623/1‑301 (MQ=255)`  
                                                                                                                       `atgatgTACTTAATGCCATTACTCATCGTCATTGTAGGATGCCTTGCGCTCCACTGTAGCGATGATGATATGCCAGATGGTCACGCTTAATACGACTCACTAAAGGAGACACTATATGTTTCGACTTCATTACAACAAAAGCGTTAAGAATTTCACGGTTCGCCGTGCTGACCGTTCAATCGTATGTGCGAGCGAGCGCCGAGCTAAGATACCTCTTATTGGTAACACAGTTCCTTTGGCACCGAGCGTCCACATCATTATCACCCGTGGTGACTTTGAGAAAGCAATAGACAAGAAACGt                                                                                                                                                                                       >  1:136105/1‑301 (MQ=255)`  
                                                                                                                        `tgatgTACTTAATGCCATTACTCATCGTCATTGTAGGATGCCTTGCGCTCCACTGTAGCGATGATGATATGCCAGATGTTCACGCTTAATACGACTCACTAAAGGAGACACTATATGTTTCGACTTCATTACAACAAAAGCGTTAAGAATTTCACGGTTCGCCGTGCTGACCGTTCAATCGTATGTGCGAGCGAGCGCCGAGCTAAGATACCTCTTATTGGTAACACAGTTCCTTTGGCACCGAGCGTCCACATCATTATCACCCGTGGTGACTTTGAGAAAGCAATAGACAAGAAACGTc                                                                                                                                                                                      <  1:298967/301‑1 (MQ=255)`  
                                                                                                                         `gatgTACTTAATGCCATTACTCATCGTCATTGTAGGATGCCTTGCGCTCCACTGTAGCGATGATGATATGCCAGATGGTCACGCTTAATACGACTCACTAAAGGAGACACTATATGTTTCGACTTCATTACAACAAAAGCGTTAAGAATTTCACGGTTCGCCGTGCTGACCGTTCAATCGTATGTGCGAGCGAGCGCCGAGCTAAGATACCTCTTATTGGTAACACAGTTCCTTTGGCACCGAGCGTCCACATCATTATCACCCGTGGTGACTTTGAGAAAGCAATAGACAAGAAACGTcc                                                                                                                                                                                     <  1:284412/301‑1 (MQ=255)`  
                                                                                                                            `gTACTTAATGCCATTACTCATCGTCATTGTAGGATGCCTTGCGCTCCACTGTAGCGATGATGATATGCCAGATGGTCACGCTTAATACGACTCACTAAAGGAGACACTATATGTTTCGACTTCATTACAACAAAAGCGTTAAGAATTTCACGGTTCGCCGTGCTGACCGTTCAATCGTATGTGCGAGCGAGCGCCGAGCTAAGATACCTCTTATTGGTAACACAGTTCCTTTGGCACCCAGCGTCCACATCATTATCACCCGTAGTGACTTTGAGAAAGCAATAGACAAGAAACGTCCggg                                                                                                                                                                                  >  1:190795/1‑300 (MQ=255)`  
                                                                                                                              `ggtataagagacagcTCATCGTCATTGTAGGATGCCTTGCGCTCCACTGTAGCGATGATGATATGCCAGATGGTCACGCTTAATACGACTCACTAAAGGAGACACTATATGTTTCGACTTCATTACAACAAAAGCGTTAAGAATTTCACGGTTCGCCGTGCTGACCGTTCAATCGTATGTGCGAGCGAGCGCCGAGCTAAGATACCTCTTATTGGTAACACAGTTCCTTTGGCACCGAGCGTCCACATCATTATCACCCGTGGTGACTTTGAGAAAGCAATAGACAAGAAACGTCCGATTc                                                                                                                                                                                <  1:64322/287‑1 (MQ=255)`  
                                                                                                                              `aCTTAATGCCATTACTCATCGTCATTGTAAGATGCCTTGCGCTCCACTGTAGCGATGGTGATATGCCAGATGGTCACGCTTAATACGACTCACTAAAGGAGACACTATATGTTTCGACTTCATTACAACAAAAGCGTTAAGAATTTCACGGTTCGCCGTGCTGACCGTTCAATCGTATGTGCGAGCGAGCGCCGAGCTAAGATACCTCTTATTGGTAACACAGTTCCTTTGGCACCGAGCGTCCACATCATTATCACCCGTGGTGACTTTGAGAAAGCAATAGACAAGAAACGTCCGGTTc                                                                                                                                                                                <  1:305998/301‑1 (MQ=255)`  
                                                                                                                               `cTTAATGCCATTACTCATCGTCATTGTAGGATGCCTTGCGCTCCACTGTAGCGATGATGATATGCCAGATGGTCACGCTTAATACGACTCACTAAAGGAGACACTATATGTTTCGACTTCATTACAACAAAAGCGTTAAGAATTTCACGGTTCGCCGTGCTGACCGTTCAATCGTATGTGCGAGCGAGCGCCGAGCTAAGATACCTCTTCTTGGTCACACAGTTCCTTTGGCACCGAGCGTCAACATCCTTATCACCCGTGGTGACTTTGCGAAAGCAATAGACAAGAAACGTCCGGTTCt                                                                                                                                                                               >  1:130490/1‑301 (MQ=255)`  
                                                                                                                               `cTTAATGCCATTACTCATCGTCATTGTAGGATGCCTTGCGCTCCACTGTAGCGATGATGATATGCCAGATGGTCACGCTTAATACGACTCACTAAAGGAGACACTATATGTTTCGACTTCATTACAACAAAAGCGTTAAGAATTTCACGGTTCGCCGTGCTGACCGTTCAATCGTATGTGCGAGCGAGCGCCGAGCTAAGATACCTCTTATTGGTAACACAGTTCCTTTGGCACCGAGCGTCCACATCATTATCACCCGTGGTGACTGTGAGAAAGCAATAGACAAGAAACGTCCGGTTCt                                                                                                                                                                               >  1:17010/1‑301 (MQ=255)`  
                                                                                                                                  `aaTGCCATTACTCATCGTCATTGTAGGATGCCTTGCGCTCCACTGTAGCGATGATGATATGCCAGATGGTCACGCTTAATACGACTCACTAAAGGGGACACTATATGTTTCGGCTTCATTACAACAAAAGCGTTAAGAATTTCACGCTTCGCCGTGCTGACCGTTCCATCGTATGTGCGAACGAGCTCCGAGCTAAGATACCTCTTATTGGTAACACAGTTCCTTTGGCACCGAGCGTCCACATCATTATCACCCGTGGCGACTTTGAGAAAGCAATAGACAAGAAACGTCCGGTTCTTAg                                                                                                                                                                            <  1:270931/301‑1 (MQ=255)`  
                                                                                                                                  `aaTGCCATTACTCATCGTCATTGTAGGATGCCTTGCGCTCCACTGTAGCGATGATGATATGCCAGATGGTCACGCTTAATACGACTCACTAAAGGAGACACTATATGTTTCGACTTCATTACAACAAAAGCGTTAAGAATTTCACGGTTCGCCGTGCTGACCGTTCAATCGTATGTGCGAGCGAGCGCCGAGCTAAGATACCTCTTATTGGTAACACAGTTCCTTTGGCACCGAGCGTCCACATCATTATCACCCGTGGTGACTTTGAGAAAGCAATAGACAAGAAACGTCCGGTTCTTAg                                                                                                                                                                            <  1:50670/301‑1 (MQ=255)`  
                                                                                                                                            `cTCATCGTCATTGTAGGATGCCTTGCGCTCCACTGTAGCGATGATGATATGCCAGATGGTCACGCTTAATACGACTCACTAAAGGAGACACTATATGTTTCGACTTCATTACAACAAAAGCGTTAAGAATTTCACGGTTCGCCGTGCTGACCGTTCAATCGTATGTGCGAGCGAGCGCCGAGCTAAGATACCTCTTATTGGTAACACAGTTCCTTTGGCACCGAGCGTCCACATCATTATCACCCGTGGTGACTTTGAGAAAGCAATAGActgtctcttatacacatctccgagcccacgg                                                                                                                                                                  >  1:255841/1‑271 (MQ=255)`  
                                                                                                                                                        `gTAGGATGCCTTGCGCTCCACTGTAGCGATGATGCTATGCCAGATGGTCACGCTTAATACGACTCACTAAAGGAGACACTATATGTTTCGACTTCATTACAACAAAAGCGTTAAGAATTTCACGGTTCGCCGTTCTGACCGTTCACTCGTATGTGCGAGCGAGCGCCGCGCTAACATACCTCTTATTGGTAACACAGTTCCTTTGGCACCCATCGTCCACAACATTCTCACACGTGGTGACTTTGAGAAAGCAATAGACAAGAAACGTCCGGTTCTTAGTGTGGCAGTGACACGCTTCCCg                                                                                                                                                      >  1:287293/1‑301 (MQ=255)`  
                                                                                                                                                        `gTAGGATGCCTTGCGCTCCACTGTAGCGATGATGATATGCCAGATGGTCACGCTTAATACGACTCACTAAAGGAGACACTATATGTTTCGACTTCATTACAACAAAAGCGTTAAGAATTTCACGGTTCGCCGTGCTGACCGTTCAATCGTATGTGCGAGCGAGCGCCGAGCTAAGATACCTCTTATTGGTAACACAGTTCCTTTGGCACCGAGCGTCCACATCATTATCACCCGTGGTGACTTTGAGAAAGCAATAGACAAGAAACGTCCGGTTCTTAgctgtctcttatacacatctccg                                                                                                                                                      >  1:85234/1‑279 (MQ=255)`  
                                                                                                                                                         `tAGGATGCCTTGCGCTCCACTGTAGCGATGATGATATGCCAGATGGTCACGCTTAATACGACTCACTAAAGGAGACACTATATGTTTCGACTTCATTACAACAAAAGCGTTAAGAATTTCACGGTTCGCCGTGCTGACCGTTCAATCGTATGTGCGAGCGAGCGCCGAGCTAAGATACCTCTTATTGGTAACACAGTTCCTTTGGCACCGAGCGTCCACATCATTATCACCCGAGGTGACTTTGAGAAAGCAATAGACAAGAAACGTCCGGTTCTTAGTGTGGCAGTGACCCGCTTCCCGt                                                                                                                                                     <  1:345167/301‑1 (MQ=255)`  
                                                                                                                                                            `gATGCCTTGCGCTCCACTGTAGCGATGATGATATGCCAGATGGTCACGCTTAATACGACTCACTAAAGGAGACACTATATGTTTCGACTTCATTACAACAAAAGCGTTAAGAATTTCACGGTTCGCCGTGCTGACCGTTCAATCGTATGTGAGAGCGAGCGCCGAGCTAAGATACCTCTTATTGGTAACACAGTTCCTTTGGCACCGTGCGTCCACATCATTATCACCCGTGGTGACTTTGAGAAAGCAATAGACAAGAAACGTCCGGTTCTTAGTGTGGCAGTGACCCGCTTCCCGTTcg                                                                                                                                                  >  1:209546/1‑301 (MQ=255)`  
                                                                                                                                                              `tGCCTTGCGCTCCACTGTAGCGATGATGATATGCCAGATGGTAACGCTTAATACGACTCACTAAAGGAGACACTATATGTTTCGACTTCATTACAACAAAAGCGTTAAGAATTTCACGGTTCACCGTGCTGACCGTTCAATCGTATGTGCGAGCGAGCGCCGAGCTAAGATACCTCTTATTGGTAACACAGTTCCTTTGGCACCGAGCGTCCACATCATTATCACCCGTGGTGACTTTGAGAAAGCAATAGACAAGAAACGTCCGGTTCTTAGTGTGGCAGTGACCCGCTTCCCGTTcgtc                                                                                                                                                <  1:328319/301‑1 (MQ=255)`  
                                                                                                                                                               `gCCTTGCGCTCCACTGTAGCGATGATGATATGCCAGATGGTCACGCTTAATACGACTCACTAAAGGAGACACTATATGTTTCGACTTCATTACAACAAAAGCGTTAAGAATTTCACGGTTCGCCGTGCTGACCGTTCAATCGTATGTGCGAGCGAGCGCCGAGCTAAGATACCTCTTATTGGTAACACAGTTCCTTTGGCACCGAGCGTCCACATCATTATCACCCGTGGTGACTTTGAGAAAGCAATAGACAAGAAACGTCCGGTTCTTAGTGTGGCAGTGACCCGCTTCCCGTTcgtcc                                                                                                                                               >  1:56055/1‑301 (MQ=255)`  
                                                                                                                                                                  `ttGCGCTCCTCTGTAGCGATGATGATATGCCAGATGGTCACGCTTAATACGACTCACTAAAGTAGACACTATATGTTTCGACTTCATTACAACAAAAGCGTTAAGAATTTCACGGTTCGCCGTGCTGACCGTTCAATCGTATGTGCGAGCGAGCGCCGAGCTAAGATACCTCTTATTGGTAACACAGTTCCTTTGGCACCGAGCGTCCACATCATTATCACCCGTGGTGACTTTGAGAAAGCAATAGACAAGAAACGTCCGGTTCTTAGTGTGGCAGTGACCCGCTTCCCGTTcgtccgtc                                                                                                                                            <  1:2909/301‑1 (MQ=255)`  
                                                                                                                                                                         `ccACTGTAGCGATGATGATATGCCAGATGGTCACGCTTAATACGACTCACTAAAGGAGACACTATATGTTTCGACTTCATTACAACAAAAGCGTTAAGAATTTCACGGTTCGCCGTGCTGACCGTTCAATCGTATGTGCGAGCGAGCGCCGAGCTAAGATACCTCTTATTGGTAACACAGTTCCTTTGGCACCGAGCGTCCACATCATTATCACCCGTGGTGACTTTGAGAAAGCAATAGACAAGAAACGTCCGGTTCTTAGTGTGGCAGTGACCCGCTTCCCGTTCGTCCGTCTGTTACt                                                                                                                                     >  1:245847/1‑301 (MQ=255)`  
                                                                                                                                                                         `ccACTGTAGCGATGATGATATGCCAAATGGTCACGCTTAATACGACTCACTAAAGGAGACACTATATGTTTCGACTTCATTACAACAAACGCGTTAAGAATTTCACGGTTCGCCGTTCTGACCGTTCAATCGTATGTGCGAGCCTGCGCCGAGCTAAGATACCTCTTATTGGTAACACAGTTCCTTTGTCACCGAGCGTCCACATCTTTATCACCCGTGGTGACTTTGAGAAAGCAATAGACAAAAAACGTCCGGTTCTTAGTGTGGAAGTGACCCTCTTCCCGTTCGTCCGTCGGTTAcc                                                                                                                                     >  1:150623/1‑300 (MQ=255)`  
                                                                                                                                                                            `cTGTAGCGATGATGATATGCCAGATGGTCACGCTTAATACGACTCACTAAAGGAGACACTATATGTTTCGACTTCATTACAACAAAAGCGTTAAGAATTTCACGGTTCGCCGTGCTGACCGTTCAATCGTATGTGCGAGCGAGCGCCGAGCTAAGATACCTCTTATTGGTAACACAGTTCCTTTGGCACCGAGCGTCCACATCATTATCACCCGTGGTGACTTTGAGAAAGCAATAGACAAGAAACGTCCGGTTCTTAGTGTGGCAGTGACCCGCTTCCCGTTCGTCCGTCTGTTACTCaa                                                                                                                                  >  1:220883/1‑301 (MQ=255)`  
                                                                                                                                                                               `aagagacagtgatATGCCAGATGGTCACGCTTAATACGACTCACTAAAGGAGACACTATATGTTTCGACTTCATTACAACAAAAGCGTTAAGAATTTCACGGTTCGCCGTGCTGACCGTTCAATCGTATGTGCGAGCGAGCGCCGAGCTAAGATACCTCTTATTGGTAACACAGTTCCTTTGGCACCGAGCGTCCACATCATTATCACCCGTGGTGACTTTGAGAAAGCAATAGACAAGAAACGTCCGGTTCTTAGTGTGGCAGTGACCCGCTTCCCGTTCGTCCGTCTGTTACTCAAACg                                                                                                                               <  1:281552/292‑1 (MQ=255)`  
                                                                                                                                                                                        `tgatATGCCAGATGGTCACGCTTAATACGACTCACTAAAGGAGACACTATATGTTTCGACTTCATTACAACAAAAGCGTTAAGAATTTCACGGTTCGCCGTGCTGACCGTTCAATCGTATGTGCGAGCGAGCGCCGAGCTAAGATACCTCTTATTGGTAACACAGTTCCTTTGGCACCGAGCGTCCACATCATTATCACCCGTGGTGACTTTGAGAAAGCAATAGACAAGAAACGTCCGGTTCTTAGTGTGGCAGTGACCCGCTTCCCGTTCGTCCGTCTGTTACTCAAACGacggtctct                                                                                                                      >  1:275984/1‑293 (MQ=255)`  
                                                                                                                                                                                        `tgatATGCCAGATGGTCACGCTTAATACGACTCACTAAAGGAGACACTATATGTTTCGACTTCATTACAACAAAAGCGTTAAGAATTTCACGGTTCGCCGTGCTGACCGTTCAATCGTATGTGCGAGCGAGCGCCGAGCTAAGATACCTCTTATTGGTAACACAGTTCCTTTGGCACCGAGCGTCCACATCATTATCACCCGTGGTGACTTTGAGAAAGCAATAGACAAGAAACGTCCGGTTCTTAGTGTGGCAGTGACCCGCTTCCCGTTCGTCCGTCTGTTACTCAAACGAATCAagga                                                                                                                      >  1:338024/1‑301 (MQ=255)`  
                                                                                                                                                                                        `tgatATGCCAGATGGTCACGCTTAAGACGACTCACTAAAGGAGAAACTATATGTTTCGACTTCATTACAACAAAAGCGTTAAGAATTTCACGGTTCGCCGTGCTGACCGTTCAATCGTATGTGCGAGCGAGCGCCGAGCTAAGATACCTCTTATTGGTAACACAGTTCCTTTGGCACCGAGCGTCCACATCATTATCACCCGTGGTGACTTTGAGAAAGCAATAGACAAGAAACGTCCGGTTCTTAGTGTGGCAGTGACCCGCTTCCCGTTCGTCCGTCTGTTACTCAAACGAATCAagga                                                                                                                      <  1:212834/301‑1 (MQ=255)`  
                                                                                                                                                                                          `atatGCCAGATGGTCACGCTTAATACGACTAACTAAAGGAGACACTAAATGTTTCGACTTCATTACAACAAAAGCGTTAAGAATTTCACGGTTCGCCGTGCTGACCGTTCAATCGTATGTGCGAGCGAGCGCCGAGCTAAGATACCTCTTATTGGTAACACAGTTCCTTTGGCACCGAGCGTCCACATCATTATCACCCGTGGTGACTTTGAGAAAGCAATAGACAAGAAACGTCCGGTTCTTAGTGTGGCAGTGACCCGCTTCCCGTTCGTCCGTCTGTTACTCAAACGAATCAaggagg                                                                                                                    <  1:308594/301‑1 (MQ=255)`  
                                                                                                                                                                                           `tatGCCAGATGGTCACGCTTAATACGACTCACTAAAGGAGACACTATATGTTTCGACTTCATTACAACAAAAGCGTTAAGAATTTCACGGTTCGCCGTGCTGACCGTTCAATCGTATGTGCGAGCGAGCGCCGAGCTAAGATACCTCTTATTGGTAACACAGTTCCTTTGGCACCGAGCGTCCACATCATTATCACCCGTGGTGACTTTGAGAAAGCAATAGACAAGAAACGTCCGGTTCTTAGTGTGGCAGTGACCCGCTTCCCGTTCGTCCGTCTGTTACTCAAACGAATCAaggaggg                                                                                                                   >  1:321823/1‑300 (MQ=255)`  
                                                                                                                                                                                                 `aGATGGTCACGCTTAATACGACTCACTAAAGGAGACACTATATGTTTCGACTTCATTACAACAAAAGCGTTAAGAATTTCACGGTTCGCCGTGCTGACCGTTCAATCGTATGTGCGAGCGAGCGCCGAGCTAAGATACCTCTTATTGGTAACACAGTTCCTTTGGAACCGAGCGTCCACATCATTATCACCCGTGGTGACTTTGAGAAAGCAATAGACAAGAAACGTCCGGTTCTTAGTGTGGCAGTGACCCGCTTCCCGTTCGTCCGTCTGTTACTCAAACGAATCAAGGAGGTGTTCTg                                                                                                             <  1:53331/301‑1 (MQ=255)`  
                                                                                                                                                                                                        `aaagCTTAATACGACTCACTAAAGGAGACATTATATGTTTCTACTTCATAACAACAAAAGAGTTAAGAATTTCACGGTTCGCCGTGCTGACCGTTCAATCGTATGTGCGAGCGAGCGCCGAGCTAAGATACCTCTTATTGGTAACACAGTTCCTTTGGCACCGAGCGTCCACATCATTATCACCCGTGGTGACTTTGAGAAAGCAATAGACAAGAAACGTCCGGTTCTTAGTGTGGCAGTGACCCGCTTCCCGTTCGTCCGTCTGTTACTCAAACGAATCAAGGAGGTGTTCTGATGGGAc                                                                                                      <  1:173576/298‑1 (MQ=255)`  
                                                                                                                                                                                                          `cGCTTAATACGACTCACTAAAGGAGACACTATATGTTTCGACTTCATTACAACAAAAGCGTTAAGAATTTCACGGTTCGCCGTGCTGACCGTTCAATCGTATGTGCGAGCGAGCGCCGAGCTAAGATACCTCTTATTGGTAACACAGTTCCTTTGGCACCGAGCGTCCACATCATTATCACCCGTGGTGACTTTGAGAAAGCAATAGACAAGAAACGTCCGGTTCTTAGTGTGGCAGTGACCCGCTTCCCGTTCGTCCGTCTGTTACTCAAACGAATCAAGGAGGTGTTCTGATGGGACTg                                                                                                    >  1:66057/1‑301 (MQ=255)`  
                                                                                                                                                                                                          `cGCTTAATACGACTCACTAAAGGAGACACTATATGTTTCGACTTCATTACAACAAAAGCGTTAAGAATTTCACGGTTCGCCGTGCTGACCGTTCAATCGTATGTGCGAGCGAGCGCCGAGCTAAGATACCTCTTATTGGTAACACAGTTCCTTTGGCACCGAGCGTCCACATCATTATCACCCGTGGTGACTTTGAGAAAGCAATAGACAAGAAACGTCCGGTTCTTAGTGTGGCAGTGACCCGCTTCCCGTTCGTCCGTCTGTTACTCAAACGAATCAAGGAGGTGTTCTGATGGGACTg                                                                                                    >  1:301964/1‑301 (MQ=255)`  
                                                                                                                                                                                                          `cGCTTAATACGACTCACTAAAGGAGACACTATATGTTTCGACTTCATTACAACAAAAGCGTTAAGAATTTCACGGTTCGCCGTGCTGACCGTTCAATCGTATGTGCGAGCGAGCGCCGAGCTAAGATACCTCTTATTGGTAACACAGTTCCTTTGGCACCGAGCGACCACATCATTATCACCCGTGGTGACTTTGAGAAAGCAATAGACAAGAAACGT‑CGGTTCTTAGTGTGGCAGTGACCCGCTTCCCGTTCGTCCGTCTGTTACTCAAACGAATCAAGGAGGTGTTCTGATGGGACTGt                                                                                                   >  1:29592/1‑301 (MQ=255)`  
                                                                                                                                                                                                          `cGCTTAATACGACTCACTAAAGGAGACACTATATGTTTCGACTTCATTACAACAAAAGCGTTAAGAATTTCACGGTTCGCCGTGATGACCGTTCAATCGTATGTGCGAGCGAGCGCCGAGCTAAGATACCTCTTATTGGTAACACAGTTCCTTTGGCACCGAGCGTCCACATCATTATCACCCGTGGTGACTTTGAGAAAGCAATAGACAAGAAACGTCCGGTTCTTAGTGTGGCAGTGACCCGCTTCCCGTTCGTCCGTCTGTTACTCAAACGAATCAAGGAGGTGTTCTGATgctgtct                                                                                                    >  1:34121/1‑295 (MQ=255)`  
                                                                                                                                                                                                          `cGCTTAATACGACTCACTAAAGGAGACACTATATGTTTCGACTTCATTACAACAAAAGCGTTAAGAATTCCACGGTTCGCCGTGCTGACCGTTCAATCGTATGTGAGAGCGAGCGCCGAGCTAAGATACCTCTTATTGGTAACACAGTTCCTTTGGCACCGAGCGTCCACATCATTATCACCCGTGGTGACTTTGAGAAAGCAATAGACAAGAAACGTCCGGTTCTTAGTGTGGCAGTGACCCGCTTCCCGTTCGTCCGTCTGTTACTCAAACGAATCAAGGAGGTGTTCTGATGGGACTg                                                                                                    >  1:266904/1‑301 (MQ=255)`  
                                                                                                                                                                                                             `gtataagagacagcTAAAGGAGACATTATATGTTTCGACTTCATTACAACAAAAGCGTTAAGAATTTCACGGTTCGCCGTGCTGACCGTTCAATCGTATGTGCGAGCGAGCGCCGAGCTAAGATACCTCTTATTGGTAACACAGTTCCTTTGGCACCGAGCGTCCACATCATTATCACCCGTGGTGACTTTGAGAAAGCAATAGACAAGAAACGTCCGGTTCTTAGTGTGGCAGTGACCCGCTTCCCGTTCGTCCGTCTGTTACTCAAACGAATCAAGGAGGTGTTCTGATGGGACTGTTa                                                                                                 <  1:3189/288‑1 (MQ=255)`  
                                                                                                                                                                                                              `tAATACGACTCACTAAAGGAGACACTATATGTTTCGACTTCATTACAACAAAAGCGTTAAGAATTTCACGGTTCGCCGTGCTGACCGTTCAATCGTATGTGCGAGCGAGCGCCGAGCTAAGATACCTCTTATTGGTAACACAGTTCCTTTGGCACCGAGCGTCCACATCATTATCACCCGTGGTGACTTTGAGAAAGCAATAGACAAGAAACGTCCGGTTCTTAGTGTGGCAGTGACCCGCTTCCCGTTCGTCCGTCTGTTACTCAAACGACTCAAGGCGGTGTTCTGATgctggctcttt                                                                                                >  1:164630/1‑291 (MQ=255)`  
                                                                                                                                                                                                              `tAATACGACTCACTAAAGGAGACACTATATGTTTCGACTTCATTACAACAAAAGCGTTAAGAATTTCACGGTTCGCCGTGCTGACCGTTCAAACGTATGTTCGAGCGAGCGCCGAGCTAAGATACCTCTTATTGGTAACACAGTTCCTTTGGCACCGAGCGTCCACATCATTATCACCCGTGGTGACTTTGAGAAAGCAATAGACAAGAAACGTCAGGTTCTTAGTGTGGCAGTGACCCGCTTCCCGTTCGTCAGTATGTTACTCAAACGAATCAAGGAGTTGTTCTTATGGGACCGttcg                                                                                                >  1:19397/1‑299 (MQ=255)`  
                                                                                                                                                                                                                `agaCGACTCACTAAAGGAGACACTATATGTTTCGACTTCATTACAACAAAAGCGTTAAGAATTTCACGGTTCGCCGTGCTGACCGTTCAATCGTATGTGCGAGCGAGCGCCGAGCTAAGATACCTCTTATTGGTAACACAGTTCCTTAGGCACCGAGCGTCCACATCATTATCACCCGTGGTGACTTTGAGAAAGCAATAGACAAGAAACGTCCGGTTCTTAGTGTGGCAGTGACCCGCTTCCCGTTCGTCCGTCTGTTACTCAAACGAATCAAGGAGGTGTTCTGATGGGACTGTTAGAt                                                                                              <  1:6899/299‑1 (MQ=255)`  
                                                                                                                                                                                                                `aTACGACTCACTAAAGGAGACACTATATGTTTCGACTTCATTACAACAAAAGCGTTAAGAATTTCACGGTTCGCCGTGCTGACCGTTCAATCGTATGTGCGAGCGAGCGCCGAGCTAAGATACCTCTTATTGGTAACACAGTTCCTTTGGCACCGAGCGTCCACATCATTATCACCCGTGGTGACTTTGAGAAAGCAATAGACAAGAAACGTCCGGTTCTTAGTGTGGCAGTGACCCGCTTCCCGTTCGTCCGTCTGTTACTCAAACGAATCAAGGAGGTGTTCTGATGGGACTGTTAGAt                                                                                              >  1:204405/1‑301 (MQ=255)`  
                                                                                                                                                                                                                  `aCGACTCACTAAAGGAGACACTATATTTTTAGACTTCATTACAACAAAAGCGTTAAGAATTTCACGGTTCGCCGTGCTGACCGTTCAATCGTATGTGCGAGCGAGCGCCGAGCTAAGATACCTCTTATTGGTAACACAGTTCCTTTGGCACCGAGCGTCCACATCATTATCACCCGTGGTGACTTTGATAAAGCAATAGACAAGAAACGTCCGGTTCTTAGTGTGGCAGTGACCCGCTTCCCGTTCGTCCGTCTGTTACTCAAACGAATCAAGGAGGTGTTCTGATGGGACTGTTAGATgg                                                                                            <  1:216862/301‑1 (MQ=255)`  
                                                                                                                                                                                                                  `aCGACTCACTAAAGGAGACACTATATGTTTCGACTTCATTACAACAAAAGCGTTAAGAATTTCACGGTTCGCCGTGCTGACCGTTCAATCGTATGTGCGAGCGAGCGCCGAGCTAAGATACCTCTTATTGGTAACACAGTGCCGTTGGCACCGAGCGTCCACATCATTATCACCCGTGGTGACTTTGAGAAAGCAATAGACAAGAAACGTCCGGTTCTTAGTGTGGCAGTGACCCGCTTCCCGTTCGTCCGTCTGTTACTCAAACGAATCAAGGAGGTGTTCTGATGGGACTGTTAGATgg                                                                                            <  1:240321/301‑1 (MQ=255)`  
                                                                                                                                                                                                                  `aCGACTAACTAAAGGAGACACTATAGGTTTCGACTTCATTACAAAAAAGGCGTCAAGAATTTCACGGTTCGCCGTGCTGACCGTTCAATCGTATGTGCGAGCGAGCGCCGAGCTAAGATACCTCTTATTGGTAACACAGTTCCTTTGGCACCGAGCGTCCACATCATTATCACCCGTGGTGACTTTGAGAAAGCAATCGACAAGAAACGTCCGGTTCTTAGTGTGGCAGTGACCCGCTTCCCGTTCGTCCGTCTGTTACTCAAACGAATCAAGGAGGTGTTCTGATGGGACTGTTAGATgg                                                                                            <  1:183330/301‑1 (MQ=255)`  
                                                                                                                                                                                                                         `aCTAAAGGAGACACTATATGTTTCGACTTCATTACAACAAAAGCGTTAAGAATTTCACGGTTCGCCGTGCTGACCGTTCAATCGTATGTGCGAGCGAGCGCCGAGCTAAGATACCTCTTATTGGTAACACAGTTCCTTTGGCACCGAGCGTCCACATCATTATCACCCGTGGTGACTTTGAGAAAGCAATAGACAAGAAACGTCCGGTTCTTAGTGTGGCAGTGACCCGCTTCCCGTTCGTCCGTCTGTTACTCAAACGAATCAAGGAGGTGTTCTGATGGGACTGTTAGATGGTGAAGcc                                                                                     <  1:82593/301‑1 (MQ=255)`  
                                                                                                                                                                                                                          `cTAAAGGAGACACTATATGTTTCGACTTCATTACAACAAAAGCGTTAAGAATTTCACGGTTCGCCGTGCTGACCGTTCAATCGTATGTGCGAGCGAGCGCCGAGCTAAGATACCTCTTATTGGTAACACAGTTCCTTTGGCACCGAGCGTCCACATCATTATCACCCGTGGTGACTTTGAGAAAGCAATAGACAAGAAACGTCCGGTTCTTAGTGTGGCAGTGACCCGCTTCCCGTTCGTCCGTCTGTTACTCAACCGAATCAAGGAGGTGTTCTGATGGGACTGTTAGATGGTGAAGccg                                                                                    >  1:176327/1‑300 (MQ=255)`  
                                                                                                                                                                                                                          `cTAAAGGAGACACTATATGTTTCGACTTCATTACAACAAAAGCGTTAAGAATTTCACGGTTCGCCGTGCTGACCGTTCAATCGTATGTGCGAGCGAGCGCCGAGCTAAGATACCTCTTATTGGTAACACAGTTCCTTTGGCACCGAGCGTCCACATCATTATCACCCGTGGTGACTTTGAGAAAGCAATAGACAAGAAACGTCCGGTTCTTAGTGTGGCAGTGACCCGCTTCCCGTTCGTCCGTCTGTTACTCAAACGAATCAAGGAGGTGTTCTGATGGGACTGTTAGATGGTTAAGCCt                                                                                    >  1:186549/1‑301 (MQ=255)`  
                                                                                                                                                                                                                            `aaaGTAGACACTATATGTTTCGACTTCATTACAACAAAAGCGTTAAGAATTTCACGGTTCGCCGTGCTGACCGTTCAATCGTATGTGCGAGCGAGCGCCGAGCTAAGATACCTCTTATTGGTAACACAGTTCCTTTGGCACCGAGTGTCCACATCATTATCACCCGTGGTGACTTTGAGAAAGCAATAGACAAGAAACGTCCGGTTCTTAGTGTGGCAGTGTCCTGCTTCCCGTTCGTCCGTCTGTTACTCAAACGAATCAAGGAGGTGTTCTGATGGGACTGTTAGATGGTGAAGCCTgg                                                                                  >  1:248491/1‑301 (MQ=255)`  
                                                                                                                                                                                                                                `gagaCACTATATGTTTCGACTTCATTACAACAAAAGCGTTAAGAATTTCACGGTTCGCCGTGCTGACCGTTCAATCGTATGTGCGAGCGAGCGCCGAGCTAAGATACCTCTTATTGGTAACACAGTTCCTTTGGCACCGAGCGTCCACATCATTATCACCCGTGGTGACTTTGAGAAAGCAATAGACAAGAAACGTCCGGTTCTTAGTGTGGCAGTGACCCGCTTCCCGTTCGTCCGTCTGTTACTCAAACGAATCAAGGAGGTGTTCTGATGGGACTGTTAGAtctgtctcttatacaca                                                                              >  1:251913/1‑285 (MQ=255)`  
                                                                                                                                                                                                                                    `cacTATATGTTTCGACTTCATTACAACAAAAGCGTTAAGAATTTCACGGTTCGCCGTGCTGACCGTTCAATCGTATGTGCGAGCGAGCGCCGAGCTAAGATACCTCTTATTGGTAACACAGTTCCTTTGGTACCGAGCGTCCACATCATTATCACCCGTGGTGACTTTGAGAAAGCAATAGACAAGAAACGTCCGGTTCTTAGTGTGGCAGTGACCCGCTTCCCGTTCGTCCGTCTGTTACTCAAACGAATCAAGGAGGTGTTCTGATGGGACTGTTAGATGGTGAAGCCTGGGAAAAAGa                                                                          >  1:223858/1‑301 (MQ=255)`  
                                                                                                                                                                                                                                    `cacTATATGTTTCGACTTCATTACAACAAAAGCGTTAAGAATTTCACGGTTCGCCGTGCTGACCGTTCAATCGTATGTGCGAGCGAGCGCCGAGCTAAGATACCTCTTATTGGTAACACAGTTCCTTTGGCACCGAGCGTCCACATCATTATCACCCGTGGTGACTTTGAGAAAGCAATAGACAAGAAACGTCCGGTTCTTAGTGTGGCAGTGACCCGCTTCCCGTTCGTCCGTCTGTTACTCAAACGAATCAAGGAGGTGTTCTGATGGGACTGTTAGATGGTGAAGCCTGGGAAAAAGa                                                                          >  1:242560/1‑301 (MQ=255)`  
                                                                                                                                                                                                                                    `cacTATATGTTTCGACTTCATTACAACAAAAGCGTTAAGAATTTCACGGTTCGCCGTGCTGACCGTTCAATCGTATGTGCGAGCGAGCGCCGAGCTAAGATACCTCTTATTGGTAACACAGTTCCTTTGGCACCGAGCGTCCACATCATTATCACCCGTGGTGACTTTGAGAAAGCAATAGACAAGAAACGTCCGGTTCTTAGTGTGGCAGTGACCCGCTTCCCGTTCGTCCGTCTGTTACTCAAACGAATCAAGGAGGTGTTCTGATGGGACTGTTAGATGGTGAAGCCTGGGAAAAAGa                                                                          >  1:109066/1‑301 (MQ=255)`  
                                                                                                                                                                                                                                      `cTATATGTTTCGACTTCATTACAACAAAAGCGTTAAGAATTTCACGGTTCGCCGTGCTGACCGTTCAATCGTATGTGCGAGCGAGCGCCGAGCTAAGATACCTCTTATTGGTAACACAGTTCCTTTGGCACCGAGCGTCCACATCATTATCACCCGTGGTGACTTTGAGAAAGCAATAGACAAGAAACGTCCGGTTCTTAGTGTGGCAGTGACCCGCTTCCCGTTCGTCTGTCTGTTACTCAAACGAATCAAGGAGGTGTTCTGATGGGACTGTTAGATGGTGAAGCCTGGGAAAAAGaaa                                                                        >  1:234538/1‑301 (MQ=255)`  
                                                                                                                                                                                                                                      `cTATATGTTTCGACTTCATTACAACAAAAGCGTTAAGAATTTCACGGTTCGCCGTGCTGACCGTTCAATCGTATGTGCGAGCGAGCGCCGAGCTAAGATACCTCTTATTGGTAACACAGTTCCTTTGGCACCGAGCGTCCACATCATTATCACCCGTGGTGACTTTGAGAAAGCAATAGACAAGAAACGTCCGGTTCTTAGTGTGGCAGTGACCCGCTTCCCGTTCGTCCGTCTGTTACTCAAACGAATCAAGGAGGTGTTCTGATGGGACTGTTAGATGGTGAAGCATGGGAAAAAGaaa                                                                        >  1:40605/1‑301 (MQ=255)`  
                                                                                                                                                                                                                                                      `cATTACAACAAAAGCGTTAAGAATTTCACGGTTCGCCGTGCTGACCGTTCAATCGTATGTGCGAGCGAGCGCCGAGCTAAGATACCTCTTATTGGTAACACAGTTCCTTTGGCACCGAGCGTCCACATCATTATCACCCGTGGTGACTTTGAGAAAGCAATAGACAAGAAACGTCCGGTTCTTAGTGTGGCAGTGACCCGCTTCCCGTTCGTCCGTCTGTTACTCCAACGAATCAAGGTGCTGTTCTGATGGGCCTGTTAGATGGTGAAGCCTGGGAAAACGCAAACCCGCCAGTACAAGc                                                        >  1:324210/1‑301 (MQ=255)`  
                                                                                                                                                                                                                                                       `aTTACAACAAAAGCGTTAAGAATTTCACGGTTCGCCGTGCTGACCGTTCAATCGTATGTGCGAGCGAGCTCCGAGCTAAGATACCTCTTATTGGTAACACAGTTCCTTTGGCACCGAGCGTCCACATCATTATCACCCGTGGTGACTTTGAGAAAGCAATAGACAAGAAACGTCCGGTTCTTAGTGTGGCAGTGACCCGCTTCCCGTTCGTCCGTCTGTTACTCAAACGAATCAAGGAGGTGTTCTGATGGGACTGTTAGATGGTGAAGCCTGGGAAAAAGAAAACCCGCCAGTACAAGCa                                                       <  1:90634/301‑1 (MQ=255)`  
                                                                                                                                                                                                                                                       `aTTACAACAAAAGCGTTAAGAATTTCACGGTTCGCCGTGCTGACCGTTCAATCGTATGTGCGAGCGAGCGCCGAGCTAAGATACCTCTTATTGGTAACACAGTTCCTTTGGCACCGAGCGTCAACATCATTATCACCCGTGGTGACTTTGAGAAAGCAATAGACAAGAAACGTCCGGTTCTTAGTGTGGCAGTGACCCGCTTCCCGTTCGTCCGTCTGTTACTCAAACGAATCAAGGAGGTGTTCTGATGGGACTGTTAGATGGTGAAGCCTGGGAAAAAGAAAACCCGCCAGTACAAGCa                                                       <  1:124507/301‑1 (MQ=255)`  
                                                                                                                                                                                                                                                                  `aGCGTTAAGAATTTCACGGTTCGCCGTGCTGACCGTTCAATCGTATGTGCGGGCGAGCGCCGAGCTAAGATACCTCTTATTGGTAACACAGTTCCTTTGGCACCGAGCGTCCACATCATTATCACCCGTGGTGACTTTGAGAAAGCAATAGACAAGAAACGTCCGGTTCTTAGTGTGGCAGTGACCCGCTTCCCGTTCGTCCGTCTGTTACTCAAACGAATCAAGGAGGTGTTCTGATGGGACTGTTAGATGGTGAAGCCTGGGAAAAAGAAAACCCGCCAGTACAAGCAACTGGGTGtat                                            <  1:273812/301‑1 (MQ=255)`  
                                                                                                                                                                                                                                                                    `gtttAAGAATTTCACGGTCCGCCGTGCTGGCCGTTCAAGCGTATGGGCGGGCGTGCGTCGAGCTAAGATACCTCTTATTGGTAACACAGTTCCTTTGGCACCGAGCGTCCACATCATTATCACCCTTGTTGACTTTGAGAAAGCAATAGACAAGAAACGTCCGGTTCTTAGTGTGGCAGTGACCCGCTTCCCGTTCGTCCGTCTGTTACTCAAACGAATCAAGGAGGTGTTCTGATGGGACTGTTAGATGGTGAAGCCTGGGAAAAAGAAAACCCGCCAGTACAAGCAACTGGGTGTATAg                                          <  1:93628/299‑1 (MQ=255)`  
                                                                                                                                                                                                                                                                     `gtgaaGAATTTCACGGTTCGCCGTGCTGAACGTTCATGCGTATGTGCGAGCGAGGGCCGAGCTAAGATACCTCTTATTGGTAACACAGTGCCTTTGGCACCGAGTGTCCACATCATTAGCACCCGTGGTGACATTGAGAAAGCAATAGACAAGAAACGTCCGGTTCTTAGTGTGGCAGTGACCCGCTTCCCGTTCGTCCGTCTGTTACTCAAACGAAGAAAGGAGGTGTTCTGATGGGACTGTTAGATGGTGAAGCCTGGGAAAAAGAAAACCCGCCAGTACAAGCAACTGGGTGTATAgc                                         <  1:260325/298‑1 (MQ=255)`  
                                                                                                                                                                                                                                                                     `gTTAAGAATTTCACGGTTCGCCGTGCTGACCGTTCAATCGTATGTGCGAGCGAGCGCCGAGCTAAGATACCTCTTATTGGTAACACAGTTCCTTTGGCACCGAGCGTCCACATCATTATCACCCGGGGTGACTTTGAGAAAGCAATAGACAAGAAACGTCCGGTTCTTAGTGTGGCAGTGACCCGCTTCCCGTTCGTCCGTCTGTTACTCAAACGAATCAAGGAGGTGTTCTGATGGGACTGTTAGATGGTGAAGCCTGGGAAAAAGAAAACCCGCCAGTACAAGCAACTGGGTGTATAgc                                         <  1:293503/301‑1 (MQ=255)`  
                                                                                                                                                                                                                                                                     `gTTAAGAATTTCACGGTTCGCCGTGCGGACCGTTCAATCGTATGGGCGAGGGAGCGCCGAGCTAAGATACCTCTTATTGGTAAAACAGTTCCTTTGGCACCAAGCGTCCACATCATTATCACCCGTGGTGACTTTGAGAAAGCAATAGACAAGAAAAGTCCGGTTCTTAGTGTGGCAGTGACCCGCTTCCCGTTCGTCCGTCTGTTACTCAAACGAATAAAGGAGGTGTTCTGATGGGACTGTTAGATGGTGAAGCCTGGGAAAAAGAAAACCCGCCAGTACAAGCAACTGGGTGTATAgc                                         <  1:231324/301‑1 (MQ=255)`  
                                                                                                                                                                                                                                                                             `tttCACGGTTCGCCGTGCTGACCGTTCAATCGTATGTGCGAGCGAGCGCCGAGCTAAGATACCTCTTATTGGTAACACAGTTCCTTTGGCACCGAGCGTCCACATCATTATCACCCGTGGTGACTTTGAGAAAGCAATAGAAAAGAAACGTCCGGTTCTTAGTGTGGCAGTGACCCGCTTCCCGTTCGTCCGTCTGTTACTCAAACGAATCAAGGAGGTGTTCTGATGGGACTGTTAGATGGTGAAGCCTGGGAAAAAGAAAACCCGCCAGTACAAGCAACTGGGTGTATAgcctgtctct                                 >  1:228335/1‑296 (MQ=255)`  
                                                                                                                                                                                                                                                                                        `gCCGTGCTGACCGTTCAATCGTATGTGCGAGCGAGCGCCGAGCTAAGATACCTCTTATTGGTAACCCAGTTCCTTTGGCACCGAGCGTCCACATCATTATCACCCGTGGTGACTTTGAGAAAGCAATAGACAAGAAACGTCCGGTTCTTAGTGTGGCAGTGACCCGCTTCCCGTTCGTCCGTCTGTTACTCAAACGAATCAAGGAGGTGTTCTGATGGGACTGTTAGATGGTGAAGCCTGGGAAAAAGAAAACCCGCCAGTACAAGCAACTGGGTGTATAGCTTGCTTAGAGAAAGATGAc                      <  1:105197/301‑1 (MQ=255)`  
                                                                                                                                                                                                                                                                                         `ccGTGCTGACCGTTCAATCGTATGTGCGAGCGAGCGCCGAGCTAAGATACCTCTTATTGGTAACACAGTTCCTTTGGCACCGAGCGTCCACATCATTATCACCCGTGGTGACTTTGAGAAAGCAATAGACAAGAAACGTCCGGTTCTTAGTGTGGCCGTGACCCGCTTCCCGTTCGTCCGGCTGTTACTCAAACGAATCAAGGAGGTGTTCTGCTGGGACTGTTAGATGGTGAAGCCTGGGAAAAAGAAAACCCGCCAGTACAAGCAACTGGGTGTATAGCTTGCTTAGAGAAAGATGAcc                     >  1:179819/1‑301 (MQ=255)`  
                                                                                                                                                                                                                                                                                           `gTGCTGACCGTTCAATCGTATGTGCGAGCGAGCGCCGAGCTAAGATACCTCTTATTGGTAACACAGTTCCTTTGGCACCGAGCGTCCACATCATTATCACCCGTGGTGACTTTGAGAAAGCAATAGACAAGAAACGTCCGGTTCTTAGTGTGGCAGTGACCCGCTTCCCGTTCGTCCGTCTGTTACTCAAACGAATCAAGGAGGTGTTCTGATGGGACTGTTAGATGGTGAAGCCTGGGCAAAAGAAAACCCGCCAGTACAAGCAACTGGGTGTATAGCTTGCTTAGAGAAAGATGACCGt                   >  1:201464/1‑301 (MQ=255)`  
                                                                                                                                                                                                                                                                                           `gTGCTGACCGTTCAATCGTATGTGCGAGCGAGCGCCGAGCTAAGATACCTCTTATTGGTAACACAGTTCCTTTGGCACCGAGCGTCCACATCATTATCACCCGTGGTGACTTTGAGAAAGCAATAGACAAGAAACGTCCGGTTCTTAGTGTGGCAGTGACCCGCTTCCCGTTCGTCCGTCTGTTACTCAAACGAATCAAGGAGGTGTTCTGATGGGACTGTTAGATGGTGAAGCCTGGGAAAAAGAAAACCCGCCAGTACAAGCAACTGGGTGTATAGCTTGCTTAGAGAAAGATGACCGt                   >  1:147370/1‑301 (MQ=255)`  
                                                                                                                                                                                                                                                                                                  `ccGTTCATTCGTATGTGCGATCGAGCGCCGAGCTAAGATACCTCTTATAGGTAACACAGTTACTTGGGCACCGAGCGTCCACATCAGTATCACCCGTGGTGACTTTGAGAAAGCAAAATACAAGAAACGGCCGGTTCTTAGTGTGGCAGAGACCCGCGTCCCATTCGTCCTTCTGTTACGCAAACGAATCAAGGAGGTGTTCTGATGGGACTGTTAGAGGGTGAAGCCAGGGAAAAAGAAAACCCGCCAGTACAAGCAACTGGGTGTATAGCTTGCTTAGAGAAAGATGACCGTTATCcac            <  1:334858/301‑1 (MQ=255)`  
                                                                                                                                                                                                                                                                                                         `aTCGTATGTGCGAGCGAGCGCCGAGCTAAGATACCTCTTATTGGTAACACAGTTCCTTTGGCACCGAGCGTCCACATCATTATCACCCGTGGTGACTTTGAGAAAGCAATAGACAAGAAACGTCCGGTTCTTAGTGTGGCAGTGACCCGCTTCCCGTTCGTCCGTCTGTTACTCAAACGAATCAAGGAGGTGTTCTGATGGGACTGTTAGATGGTGAAGCCTGGGAAAAAGAAAACCCGCCAGTACAAGCAACTGGGTGTATAGCTTGCTTAGAGAAAGATGACCGTTATCCACACAccct     >  1:331202/1‑299 (MQ=255)`  
                                                                                                                                                                                                                                                                                                            `gTATGTGCGAGCGAGCGCCGAGCTAAGATACCTCTTATTGGTAACACAGTTCCTTTGGCACCGAGCGTCCACATCATTATCACCCGTGGTGACTTTGAGAAAGCAATAGACAAGAAACGTCCGGTTCTTAGTGTTGCAGTGACCCGCTTCCCGTTCGTCCGTCTGTTACTCAAACGAATCAAGGAGGTGTTCTGATGGGACTGTTAGATGGTGAAGCCTGGGAAAAAGAAAACCCGCCAGTACAAGCAACTGGGTGTATAGCTTGCTTAGAGAAAGATGACCGTTATCCACACACCTGTaa  <  1:58276/301‑1 (MQ=255)`  
                                                                                                                                                                                                                                                                                                            `gTATGTGCGAGCGAGCGCCGAGCTAAGATACCTCTTATTGGTAACACAGTTCCTTGGGCACCGAGCGTCCACATCATTATCACCCGTGGTGACTTTGAGAAAGCAATAGACAAGAAACGTCCGGTTCTTAGTGTGGCAGTGACCCGCTTCCCGTTCGTCCGTCTGTTACTCAAACGAATCAAGGAGGTGTTCTGATGGGACTGTTAGATGGTGAAGCCTGGGAAAAAGAAAACCCGCCAGTACAAGCAACTGGGTGTATAGGTTGCTTAGAGAAAGATGACCGTAATCCACACACATGTaa  >  1:118400/1‑301 (MQ=255)`  

                                                                                                                                                                                                                                                                                                            `|`                                                                                                                                                                                                                                                                                                              
`TTGCGGTATACCCTCAAGTAGCACTAGTAGTAGTTGGCGCTTGTTACTTAGCGGCAGTGTGTGCTTGCGTGTGGAGTATAGTTAACTGGTAATACGACTCACTAAAGGAGGTACACACCATGATGTACTTAATGCCATTACTCATCGTCATTGTAGGATGCCTTGCGCTCCACTGTAGCGATGATGATATGCCAGATGGTCACGCTTAATACGACTCACTAAAGGAGACACTATATGTTTCGACTTCATTACAACAAAAGCGTTAAGAATTTCACGGTTCGCCGTGCTGACCGTTCAATCGTATGTGCGAGCGAGCGCCGAGCTAAGATACCTCTTATTGGTAACACAGTTCCTTTGGCACCGAGCGTCCACATCATTATCACCCGTGGTGACTTTGAGAAAGCAATAGACAAGAAACGTCCGGTTCTTAGTGTGGCAGTGACCCGCTTCCCGTTCGTCCGTCTGTTACTCAAACGAATCAAGGAGGTGTTCTGATGGGACTGTTAGATGGTGAAGCCTGGGAAAAAGAAAACCCGCCAGTACAAGCAACTGGGTGTATAGCTTGCTTAGAGAAAGATGACCGTTATCCACACACCTGTAA  >  NC_001604.1/7672‑8272`

  

Alignment Legend

Aligned base mismatch/match (shaded by quality score): `ATCG/ATCG` `< 3 ≤` `ATCG/ATCG` `< 10 ≤` `ATCG/ATCG` `< 22 ≤` `ATCG/ATCG` `< 34 ≤` `ATCG/ATCG` `< 38 ≤` `ATCG/ATCG`

Unaligned base: `atcg`    Masked matching base: `atcg`    Alignment gap: `‑`    Deleted base: `‑`

Reads not counted as support for junction

read\_name Not counted due to insufficient overlap past the breakpoint.

read\_name Not counted due to not crossing MOB target site duplication.
